# Real-space observation of surface structuring induced by ultra-fast-laser illumination far below the melting threshold

**DOI:** 10.1038/s41598-021-91894-w

**Published:** 2021-06-24

**Authors:** Ch. Zaum, N. Osterloh, R. Darkins, D. M. Duffy, K. Morgenstern

**Affiliations:** 1 Abteilung für atomare und molekulare Strukturen (ATMOS), Institut für Festkörperphysik, Leibniz Universität Hannover, Appelstr. 2, 30167 Hannover, Germany; 2 Lehrstuhl für physikalische Chemie I, Ruhr-Universität Bochum, 44780 Bochum, Germany; 3 Department of Physics and Astronomy and London Centre for Nanotechnology, University College London, Gower Street, London, WC1E6BT UK

**Keywords:** Scanning probe microscopy, Nanoparticles, Ultrafast lasers, Surfaces, interfaces and thin films, Chemical physics

## Abstract

Intense short laser pulses are an intriguing tool for tailoring surface properties via ultra-fast melting of the surface layer of an irradiated target. Despite extensive studies on the interaction of femto-second laser interaction with matter, the initial steps of the morphological changes are not yet fully understood. Here, we reveal that substantial surface structure changes occur at energy densities far below the melting threshold. By using low-temperature scanning tunneling microscopy we resolve atomic-scale changes, i.e. the creation of nanosized adatom and vacancy clusters. The two cluster types have distinct non-linear fluence-dependencies. A theoretical analysis reveals their creation and motion to be non-thermal in nature. The formation of these atomistic changes, individually resolved here for the first time, recast our understanding of how surfaces respond to low-intensity ultra-short laser illumination. A visualization and control of the initial morphological changes upon laser illumination are not only of fundamental interest, but pave the way for the designing material properties through surface structuring.

## Introduction

Femtosecond (fs) laser-material interactions were largely investigated in the past^1,2^ because they induce unique morphological changes^3,4^ that often drastically alter a material’s physical properties^5^. Novel effects include superhydrophilic, superhydrophobic, and multifunctional surfaces^6,7^. Such functionalized surfaces have found a wide range of applications that can also be employed industrially^8^. However, a clear understanding of even the simplest morphological responses to ultra-fast laser illumination is still lacking because an atomistic visualization of the structural changes to a metal during ultra-fast structural dynamics has not yet been achieved experimentally.

From a scientific viewpoint, the investigation of a material’s response to femtosecond laser illumination provides unparalleled insight into material behavior far from equilibrium under the extreme conditions of ultra-high peak power and ultra-short pulse duration. These processes are well understood only for high laser fluences (=energy per pulse and unit area) above the melting and ablation thresholds, where the surface damage results from heat-induced stress confinement^2^, amongst others. For these fluences, micro-scale imaging revealed the importance of visualizing the induced changes in real-space, as captured in time-delayed experiments^9–11^. In contrast, understanding the processes happening at or below these thresholds is much more demanding as they require methods with nanoscale resolution. For instance, subsurface cavities of sub-micron size are created for aluminum^12^ and for Ag(100)^13^ just below their ablation threshold at $\approx 65~\hbox {mJ/cm}^2$ and $85~\hbox {mJ/cm}^2$ absorbed fluences, respectively.

On the other hand, ultra-short laser-induced reactions, performed at lower absorbed single-pulse fluences of around $1~\hbox {mJ/cm}^2$, assume a static surface^14^. It is well-established that such femto-chemical reactions are driven by electronic excitations of the adsorbates through a hot electron gas induced via laser absorption by the electrons of the metal. Here the diverse exponents of a characteristic non-linear fluence dependence on the reaction yield, ranging from 2 to 15^15^, remains to be explained. To our knowledge, a surface restructuring induced by femto-second laser illumination in this range of fluences has not yet been reported.

Here, we reveal via low-temperature scanning tunneling microscopy the response of a Ag(100) surface to a train of ultra-short laser pulses at incoming (absorbed) single-pulse fluences below 5.4 $(0.315)~\hbox {mJ/cm}^2$, i.e. far below reported ablation threshold for absorbed fluences in Ag between $85~\hbox {mJ/cm}^2$^13^ and $\approx 1.5~\hbox {J/cm}^2$ for a single shot^16^. Tailoring such structures could pave the way for a design of surfaces with wanted properties. A high-quality real-space imaging allows us to resolve, for the first time, laser-induced morphological surface structure formation in metals on the atomic-scale. Our precise characterization of how this illumination modifies the surface suggests severe surface changes at fluences that are too low to break inter-atomic bonds.

## Methods

The measurements are performed with the aid of a unique set-up that features direct optical access to the sample to align a femto-second laser spot with the tunneling gap of a low-temperature scanning tunneling microscope (STM) operated under ultra-high vacuum^17^.

The Ag(100) surface is cleaned by repeated cycles of $\hbox {Ne}^+$ sputtering and annealing. For all but a control experiment, the sample is subsequently cooled to 100 K, at which a mild sputtering pulse (0.5 keV, $0.014~\upmu \hbox {A}$, 3 s) is given. Directly after the pulse, the sample is cooled to $\approx ~50~\hbox {K}$ and transferred into the STM, where measurements are performed at 7 K.

**Figure 1:**
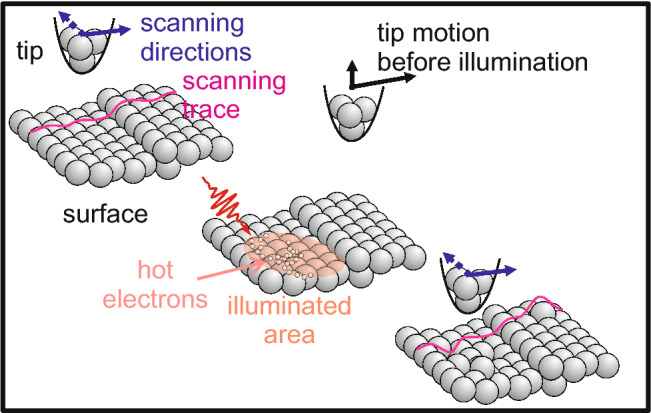
Schematics of the experiment.

An experiment starts by recording several STM images of the pristine or sputtered surface (Fig. 1). Having the tip grounded and retracted by more than $1~\upmu \hbox {m}$ from the scanned area and the incoming laser light, the sample is exposed to a train of $10^7$ laser pulses at a repetition rate of 250 kHz. The frequency-doubled commercial laser (REGA 9050 from Coherent) provides 400 nm pulses with ($72\pm 2$) fs. Note that the tip resides in the periphery of the laser profile during illumination, yet without an applied bias. As field enhancement is restricted to approximately 10 nm around the tip for our laser parameters and tip radii^18^, the tip does not alter the laser field in the imaged area. The laser energy is absorbed by the electrons of the metallic surface leading to a so-called hot electron gas^14^. To determine the changes to the sample surface by these hot electrons, the same region of the surface is imaged after illumination at tunneling parameters, which were ensured not to alter the surface during imaging. For a quantitative determination of reaction rates, it is not only essential to image exactly the same spot of the surface before and after laser illumination, but also to align the tip to the maximum of the laser spot profile.

### Tip alignment through photo-electron signal

**Figure 2:**
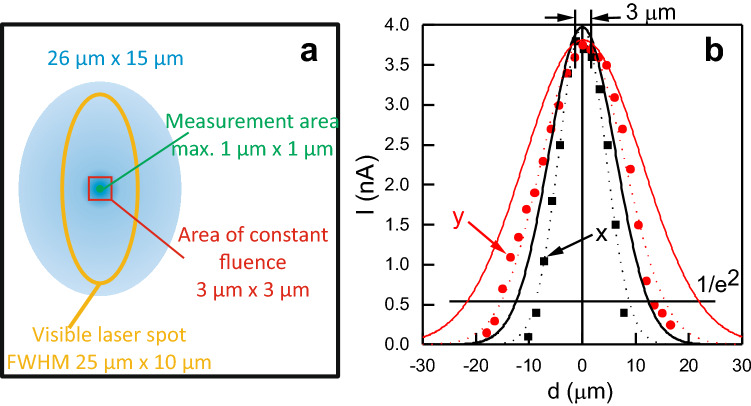
Adjustment of laser spot into tunneling gap: (**a**) Schematics (**b**) Dependence of photo-electron current *I* on lateral position *d* on the sample within the laser spot for an absorbed SPP fluence of $297~\upmu \hbox {J/cm}^2$; incoming SPP fluence: $5.32~\hbox {mJ/cm}^2$, $10^7$ pulses; 72 fs; thin dotted lines are Gaussian fits to the data and thick lines the square root of these fits.

The laser light is focused *in-situ* below the tip. The $65^\circ$ incidence angle of the laser leads to an elliptical spot, which has been characterized optically before to be around $25~\upmu \hbox {m}$ by $10~\upmu \hbox {m}$ by moving the laser spot across the tip apex, which serves as a slit, as described in detail in^17^ (Fig. 2a). Here, we use the tip as a photo-electron detector to determine the profile of the laser spot more precisely. For this aim, the photo-electron signal is spatially resolved in two perpendicular directions by moving the tip across the laser spot (Fig. 2b). The y-signal is somewhat broader than the x-signal. Both show the expected Gaussian profile of a laser spot.

At an energy of 3.1 eV per photon, a single photon is not sufficient to overcome the work function of Ag(100) reported as 4.22 eV^19^ or 4.43 eV^20^. Thus, the square root of the measured signal corresponds to the single-photon laser profile. From this procedure we infer a width of the elliptical spot of $\sim ~43~\upmu \hbox {m}$ by $\sim 25~\upmu \hbox {m}$ at its $1/e^2$ value and of $\sim 26~\upmu \hbox {m}$ and $\sim 15~\upmu \hbox {m}$ at half values, Fig. 2b, giving for quantification a more precise value than the optical signal. The thus determined size of the laser spot at the sample is used to calculate the fluence of an experiment from the measured power at a position equivalent to the one of the sample. The fluences given below are the absorbed peak fluences for a single pulse. These values consider that the absorption of Ag(100) is 5.6% for 400 nm and *p*-polarized light at the $65^\circ$ incident angle used here^21^. For the experiments on Cu, the corresponding absorption of 72% is utilized. These absorbed single-pulse peak (SPP) fluences correspond to twice the average fluences within the $1/\hbox {e}^2$ diameter of the laser spot conventionally reported in femtochemistry, where the chemistry across the entire laser-spot is monitored by averaging methods. In contrast STM images are recorded within a small region below $1~\upmu \hbox {m}$ by $1~\upmu \hbox {m}$ (Fig. 2a). At the top of the Gaussian profile, the local fluence varies only by around 4% over an even larger range of $3~\upmu \hbox {m}$ by $3~\upmu \hbox {m}$ (Fig. 2b).

Most importantly, we use this photo-electron signal to align the tip more precisely than optically feasible during the experiments along the profile line of the laser spot, a procedure needed in particular for quantitative comparison.

### DFT calculations

The DFT calculations of Ag(100) were performed using Quantum Espresso^22^. A $(3\times 3)$ periodicity was employed laterally and the crystal was composed of four atomic layers in height with the bottom layer frozen. Artificial periodicity was applied in all three dimensions with a 1 nm vacuum slab exposing the surfaces. The GGA-PBE functional^23^ was employed with PAW pseudo-potentials. A $16^3$ Monkhorst-Pack *k*-point grid was used to integrate the Brillouin zone. A finite electronic temperature was introduced by applying a Fermi-Dirac distribution to the electronic states according to the formalism of Mermin^24^. As the electrons can take a few hundred fs to thermalise at our parameters and the low electronic temperature range of relevance to our study, the non-thermal electronic system consistently couples less strongly to the phononic system^25^. Thus, the calculated phonon temperature, predicted under the assumption of instant thermalisation, represents an upper bound.

## Results and discussion

**Figure 3:**
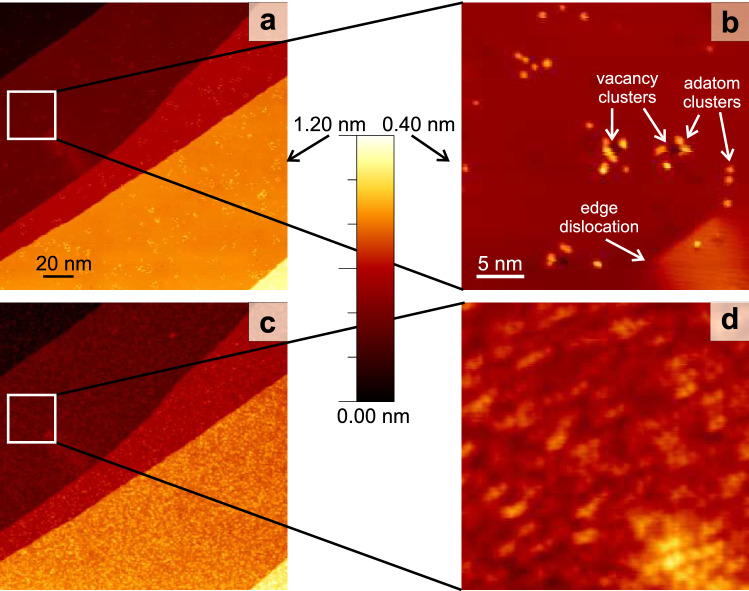
Laser-induced changes: surface before (**a**,**b**) and after (**c**,**d**) illumination with $290~\upmu \hbox {J/cm}^2$ absorbed SPP fluence, $10^7$ pulses; (**a**,**c**) overview images (**b**,**d**) zoom into squares marked in (a,c); $\hbox {V}_t=113~\hbox {mV}$, $\hbox {I}_t= 55~\hbox {pA}$, on false-color scale as indicated.

For an easier identification of the same spot of the surface, the surface is pre-structured by a sputter pulse at low temperature prior to the laser illumination (see “Methods” section). After the sputter pulse, the surface exhibits holes and protrusions that were identified before as small vacancy and adatoms clusters, respectively (Fig. 3a,b)^26–29^. An ion impact results in one vacancy cluster that is surrounded by several adatom clusters, i.e. more adatom clusters than vacancy clusters are formed. Apart from four intrinsic surface steps, there are two vaults in the overview image in Fig. 3a. Similar vaults on Ag(111) were identified as split edge dislocations^30^, caused by subsurface noble gas bubbles^31^. Here, such edge dislocations facilitate the exact superposition of images taken before and after laser illumination.

Such a surface is now exposed to ultra-fast laser light at fluences far below the ablation threshold, stated above. Upon exposing the surface to a train of $10^7$ laser pulses, at around $300~\upmu \hbox {J/cm}^2$ absorbed SPP fluence, the surface is severely altered (Fig. 3c). The roughened surface consists of adatom clusters of various sizes (Fig. 3d). Comparison of step edges before and after illumination (Fig. 3a,c) reveals that they are not altered, as confirmed on other surfaces (see below). Different positions of the tip during the laser illumination further exclude that the material originates from the tip.

**Figure 4:**
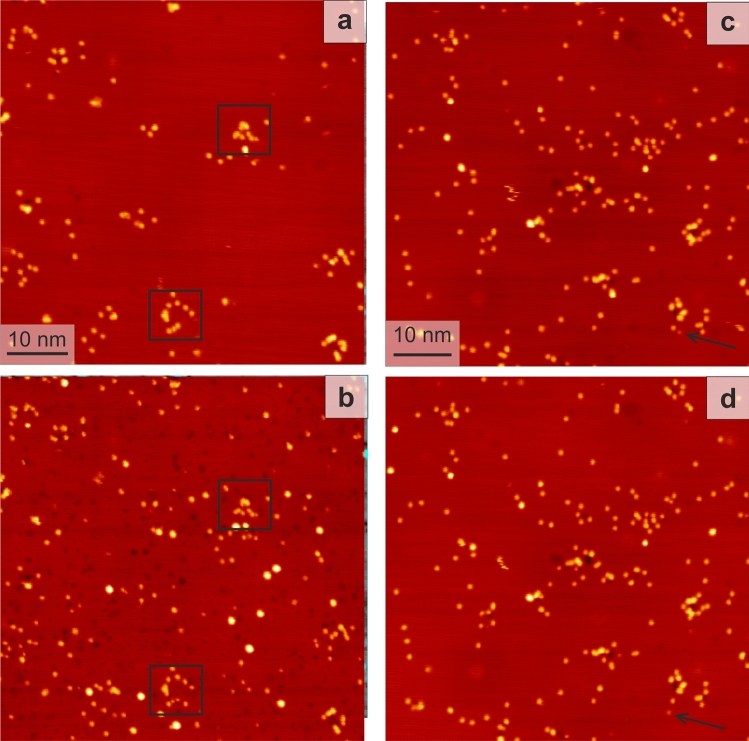
Laser-induced surface restructuring; 113 mV, 81 pA, $10^7$ pulses: surface before (**a**,**c**) and after (**b**,**d**) laser illumination with (**a**,**b**) 72 fs, absorbed SPP fluence $130~\upmu \hbox {J/cm}^2$ and (c,d) 250 fs, ${168}~\upmu \hbox {J/cm}^2$; squares in (**a**,**b**) mark two sputter damages for reference; arrows in (**c**,**d**) mark a single moving adatom.

To unravel the source of the additional material, we repeat the experiment at around half the absorbed SPP fluence (Fig. 4). As expected, roughening is less pronounced at this lower fluence. Apart from adatom clusters, also vacancy clusters cover the surface. These lower fluence results suggest that the layer of clusters observed at ${300}~\upmu \hbox {J/cm}^2$ covers a porous surface (Fig. 3). In contrast to the sputtering damage, there is no clear correlation between the positions of the adatom clusters and of the vacancy clusters. Moreover, more vacancy clusters than adatom clusters are created, with a larger variance in size.

How can the laser illumination lead to this surface modification? For the much higher SPP fluence used in^2^, the heat-induced stress confinement in the bulk altered the surface. If the process was also heat-induced here, it should persist for pulse lengths up to the picosecond range.

A laser pulse stretched to three times its original length by placing a 50 mm glass (N-BK7) into the beam does not lead to any additional adatom or vacancy clusters at a comparable SPP fluence (Fig. 4c,d). Only a few clusters change their position slightly between Fig. 4c,d, indicating an onset of phonon-driven, that is heat-induced, motion. The complete absence of additional clusters at an even slightly larger energy density than in the experiment presented in Fig. 4a,b demonstrates that the surface modification there is not related to thermal heating by the laser. It must therefore be electron-driven based on the following reasoning. For a laser illumination that is not in resonance with the adsorbate levels, the electrons of the metal absorb the photon energy^14^. A transfer of the electron energy to the lattice on the ps-time scale could heat it transiently to temperatures that are sufficient to induce changes thermally. In this case, the process should, at the same absorbed fluence, be independent of the pulse length of the laser up to the time of nuclear motion, i.e. ps. Alternately, the optically excited electrons could induce the observed processes directly, either in a single-electron or in a multi-electron process. For the latter process, the reaction yield depends on the pulse length, because the number of simultaneously available electrons decreases with increasing pulse-length. Furthermore, this number increases non-linearly with absorbed fluence^14^.

### Fluence dependence of surface modification

**Figure 5:**
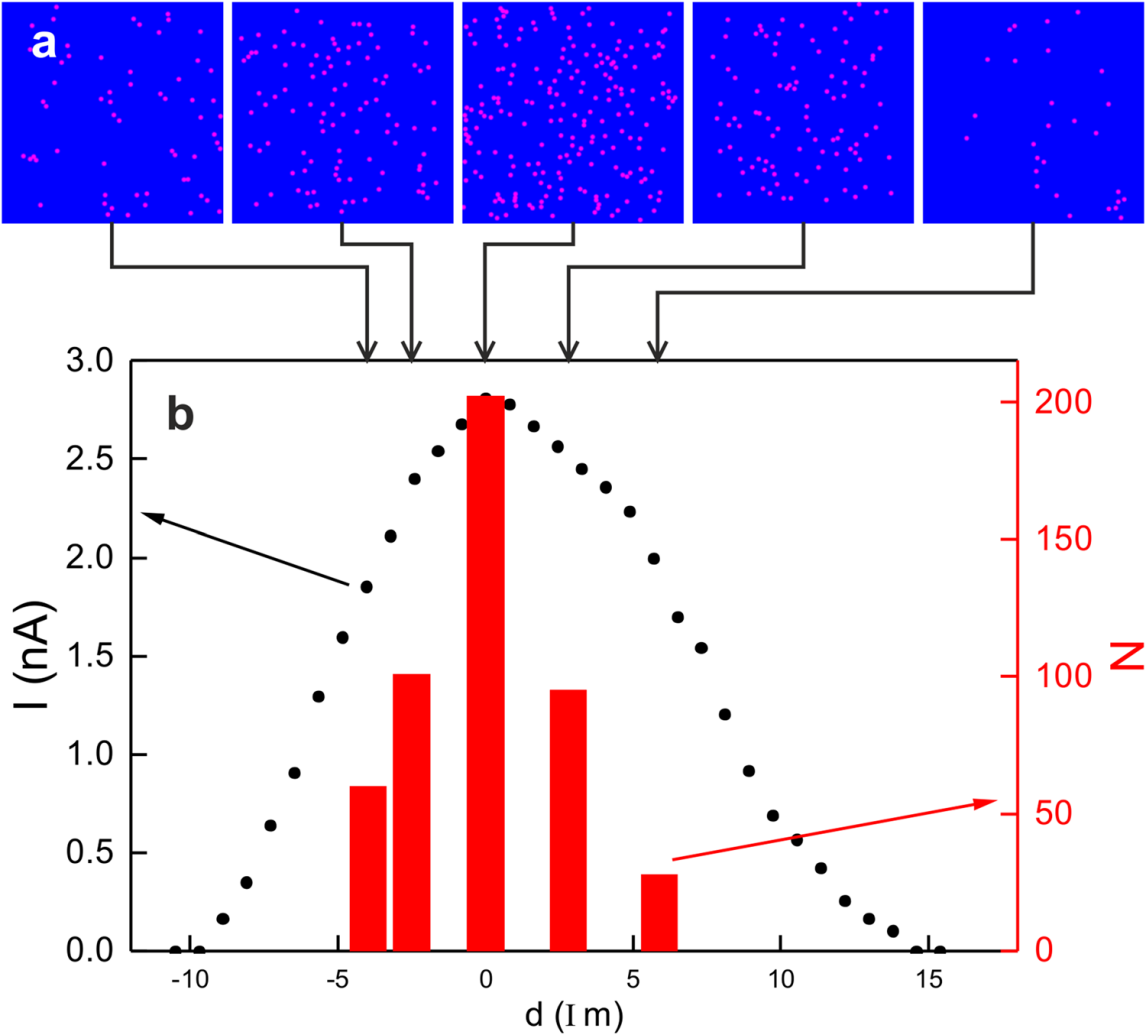
Spatial dependence of surface modification on Ag(100), $10^7$ pulses: (**a**) difference of clusters of both types as extracted from STM images before and after illumination by subtraction of the two STM images; the indicated position within the laser spot profile are determined via the photoelectron signal, collected by the STM tip (**b**) Dependence of the number of laser-induced clusters *N* of both types (bars, right axis) and photo-electron signal *I* (dots, left axis) on position within the laser spot *d*; incoming SPP fluence of $2.73~\hbox {mJ/cm}^2$ and absorbed SPP fluence of $153~\upmu \hbox {J/cm}^2$.

To elucidate this electron-driven process, we compare STM images recorded at different positions within the laser spot, which represent different local fluences (Fig. 5a). The number of additional clusters decreases rapidly off focus. A shift by $2.5~\upmu \hbox {m}$ halves and by less than $6~\upmu \hbox {m}$ decreases the number of clusters by a factor of seven. The reduction of the calculated number of available photo-electron by only 15% across this range (Fig. 5b) suggests a non-linearity of the surface modification.

**Figure 6:**
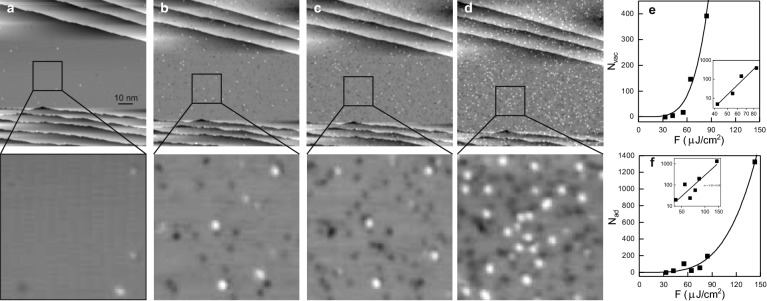
Fluence dependence: (**a**–**d**) Series of laser illuminations of the same surface region with increasing fluence; lower row: Zoom-in into the square marked in the upper row at enhanced contrast; $10^7$ pulses at absorbed SPP fluences: (**a**,**b**) ${43}~\upmu \hbox {J/cm}^2$ (**b**,**c**) ${63}~\upmu \hbox {J/cm}^2$ (**c**,**d**) ${136}~\upmu \hbox {J/cm}^2$; $\hbox {V}_t=130~\hbox {mV}$, $\hbox {I}_t = 55~\hbox {pA}$[Note that the laser alignment to the tip-sample region, though performed at much reduced fluence, leads already to the formation of a minor number of defects.]; (**e**,**f**) newly formed clusters $\Delta \hbox {N}_i$ per $1000~\hbox {nm}^2$, insets: same data on double-logarithmic scale with apparent linear fit: (**e**) vacancies (**f**) adatoms.

This non-linearity is further demonstrated by illuminating the same spot of the sample with increasing laser power, starting with an (almost) pristine surface (Fig. 6a–d). Note that this experiment demonstrates that the surface modification does not rely on the pre-existence of sputter structures, used for reference in the other experiments. In Fig. 6, considerably more vacancy clusters than adatom clusters are formed up to an absorbed SPP fluence of $60~\upmu \hbox {J/cm}^2$ (Fig. 6b,c). Adatom clusters are more numerous only above an absorbed SPP fluence of $\sim 135~\upmu ~\hbox {J/cm}^2$ (Fig. 6d). Evidently, the density of adatom and vacancy clusters depends on laser fluence.

We therefore determine the numbers separately in experiments that begin with the sputtered-only surface for each fluence. As observed qualitatively above, a small quantity of vacancy and adatom clusters form above $\sim 60~\upmu \hbox {J/cm}^2$ absorbed SPP fluence (Fig. 6e,f). Despite a marked increase in vacancy clusters above $110~\upmu \hbox {J/cm}^2$, the number of additional adatom clusters decreases at this fluence. The number of adatom clusters increases substantially only above $\sim {170}~\upmu \hbox {J/cm}^2$. A comparison of size distributions indicates that interatomic bonds may be broken at this fluence (see^15^). The double-logarithmic representation of the data suggests that $\Delta N \propto F^x$. The exponents resulting from fitting this equation to the two data sets are, at $x_{vac} = 3.9\pm 0.4$ and $x_{ad} = 4.7\pm 0.5$, well within the range of fluence-dependent exponents observed in femtochemistry, where the same large number of pulses is given at different fluences^14,15^.

Though the formation of the adatom and vacancy clusters should be intimately connected, the thresholds for a substantial formation of these two types of clusters on the surface differ by around $60~\upmu \hbox {J/cm}^2$. Such a behavior could be understood if vacancy-interstitial pairs were mostly formed subsurface, invisible to the STM, and the motion of the two species to the surface differed. Indeed, interstitials and vacancies have distinct bulk diffusion energies of 0.09 eV and 0.66 eV, respectively^32^. The large difference in their energies makes it unlikely that the laser activates them both thermally to diffuse to the surface. Moreover, for a thermally induced diffusion, the interstitial should be much more mobile than the vacancies and thus arrive first at the surface, in contrast to the experimental observation.

### Laser-surface interaction

**Figure 7:**
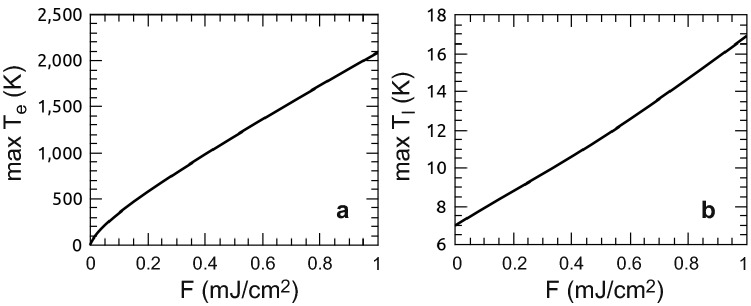
Fluence dependence of maximum temperatures reached in Ag for a 72 fs pulse at an adsorbed fluence of $1~\hbox {mJ/cm}^2$: (**a**) electron temperature $T_e$ (**b**) phonon temperature $T_l$.

Nevertheless, we explore whether the motion of the particles to the surface is thermal or electron-driven by calculating the average electronic and phononic temperatures after a short-pulse laser and their evolution in time via a one-dimensional two-temperature model^33^. This model assumes that the laser energy is absorbed fully by the electrons that equilibrate during the duration of the pulse to a Fermi-like energy distribution (described by a temperature), while the lattice stays cold. The electron and phonon systems subsequently exchange energy and move towards equilibrium, while the heat also diffuses spatially into the semi-infinite crystal. The model is applicable as long as the electron distribution equilibrates within the pulse duration and assumes this equilibration to be instantaneous. If the laser spans a sufficiently large lateral dimension, the system is treated one-dimensionally, and it is assumed that the crystal is infinitely deep. The two temperatures are calculated based on the following equations^33^:

1 $$\begin{aligned}&C_e\frac{\partial T_e}{\partial t}=\nabla \cdot (\kappa _e \nabla T_e)-G(T_e-T_l)+Q \end{aligned}$$

2 $$\begin{aligned}&C_l\frac{\partial T_l}{\partial t}=G(T_e-T_l) \end{aligned}$$

3 $$Q(z,t) = \frac{2}{{\sqrt {\pi /\log (2)} }}\frac{F}{{t_{{FWHM}} }}\alpha \exp ( - 4\log (2)(t/t_{{FWHM}} )^{2} - z\alpha )$$

where $T_e$ and $T_l$ are the electronic and lattice temperature, respectively, $C_i$ are the corresponding specific heat capacities, *G* is the electron–phonon coupling constant, *Q* is the heat source, *F* is the absorbed SPP fluence, $t_{FWHM}$ is the pulse length (FWHM), and $1/\alpha$ is the optical penetration depth. The equations were solved numerically, using the Euler method, on a grid that spanned $10~\upmu \hbox {m}$ with a 10 nm-bin size and a time integration time-step of 0.01 fs. The surface was modeled as a reflecting boundary while the other end was absorbing (implemented using Green’s method^34^). For an estimation of the highest reachable temperature, we neglect ballistic electrons, which were shown to be important for a quantitative modeling of noble metals^35^. These would reduce the maximum temperature by removing energy from the excitation region.

For the $t_{FWHM} = 72~\hbox {fs}$, utilizing for the specific heat $C_e$ and the electron–phonon coupling *G* parameters from^36^, the $T_e$-dependent functions are obtained by DFT calculations^37^. For reference, an absorbed $1~\hbox {mJ/cm}^2$ fluence with a 72 fs pulse width induces a peak electron temperature of 2100 K and a peak phonon temperature of 17 K (Fig. 7a,b). These numbers are expected to serve as safe upper bounds on the experimental values, and yet the phonon temperature is not sufficient to induce any measurable amount of interstitial or vacancy diffusion in the bulk.

### Motion from the bulk to the surface

In this section, we consider the hypothesis that interstitial and vacancies already permeate the bulk of the crystal and that the irradiation drives them to diffuse to the surface. Consider a bulk interstitial or vacancy. Its diffusion coefficient can be written as a function of time *t* and depth *z* below the surface:

4 $$\begin{aligned} D(z,t)=D_0 exp(-E_a/k_B T_l(z,t)) \end{aligned}$$

where $T_l(z,t)$ is calculated from the two-temperature model. In the case of interstitials, it was computed from molecular dynamics that $D_0 = 0.97~\hbox {nm}^2/\hbox {ps}$ and $E_a = 0.09~\hbox {eV}$. For single and double vacancies the activation energies are $E_a = 0.66~\hbox {eV}$ and $E_a = 0.56~\hbox {eV}$, respectively^32^. We here calculate the critical SPP fluence $F_c$ necessary to make either of the two particles move for a given activation energy $E_a$. The mean diffusion coefficient near to the surface at $z = 0$, where there would be the largest movement, is

5 $$\begin{aligned} \langle D \rangle =\frac{1}{t_p}\int _{0}^{t_p}{D(z=0,t)dt} \end{aligned}$$

where $t_p= 4\cdot 10^{-6}~\hbox {s}$ is the interval between consecutive pulses and the illumination lasts for $t_{total} = 40~\hbox {s}$. With the smallest possible movement taken as $\Delta z \approx 0.1~\hbox {nm}$, we calculate the critical SPP fluence $F_c$ using the relation $\langle \Delta z^2 \rangle = 2 \langle D\rangle t_{total}$. This is a robust measure of $F_c$ as scaling $\Delta z$ by a factor of ten shifts $F_c$ merely by $0.1~\hbox {mJ/cm}^2$.

**Figure 8:**
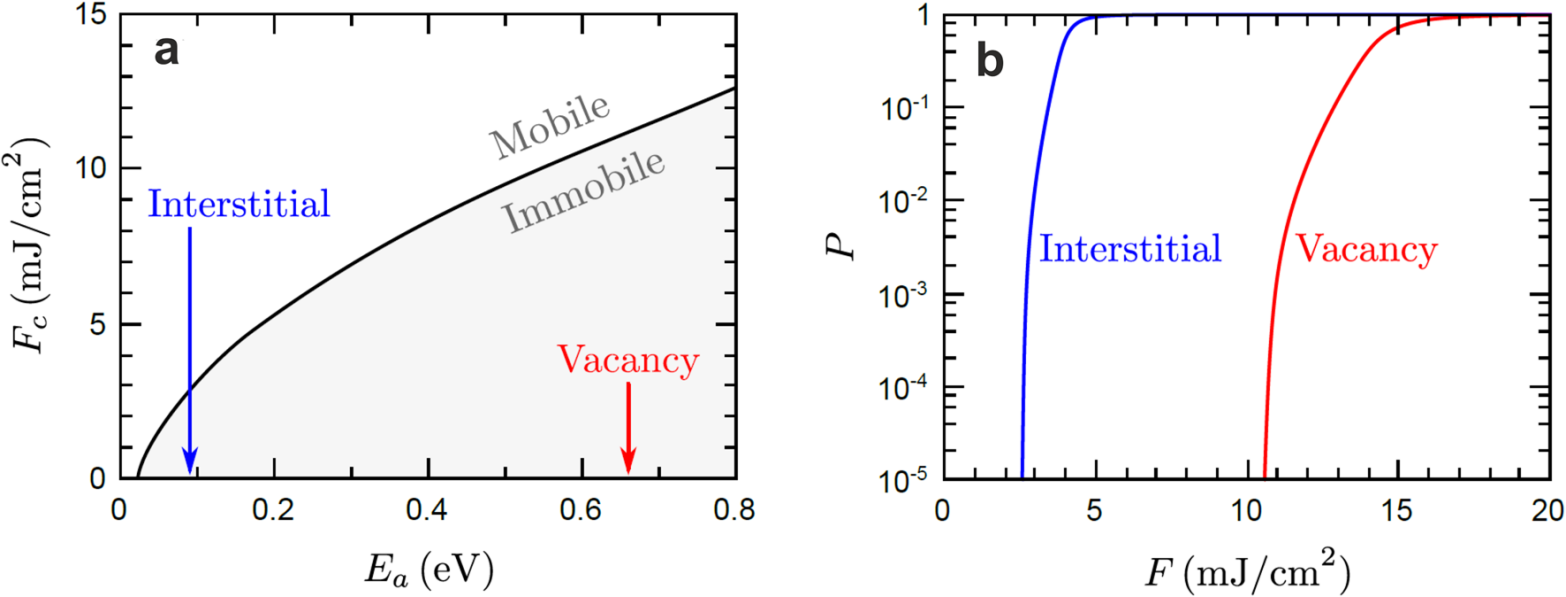
Critical fluence for bulk diffusion: (**a**) The critical absorbed SPP fluence $F_c$ required to induce thermal bulk diffusion of an interstitial or vacancy as a function of the activation energy $E_a$ associated with moving said particle (**b**) The fraction of particles within a small subsurface layer expected to diffuse to the surface over the course of the experiment as a function of the absorbed SPP fluence.

For the experimental SPP fluence range $F \le 1~\hbox {mJ/cm}^2$, the interstitials and vacancies will only move if their activation energies are below about 0.04 eV (Fig. 8a). Indeed, to move the interstitials or the vacancies would require a SPP fluence of around $2.8~\hbox {mJ/cm}^2$ or $10~\hbox {mJ/cm}^2$, respectively for an experiment employing $10^6$ pulse (Fig. 8b), i.e. an order of magnitude larger than employed in the experiment. Thus, the hot electrons are responsible for the transport of the particles to the surface, consistent with our control experiment (Fig. 4c,d) and the power-law fluence dependence of their number. Likewise, the formation of vacancy-interstitial pairs is impossible at a temperature as low as 17 K.

We infer that the lower threshold at $\sim 55~\upmu \hbox {J/cm}^2$ corresponds to the threshold for the non-thermal formation of vacancy-interstitial pairs, while the two different thresholds for the two particles at $\sim 110$ and $\sim 170~\upmu \hbox {J/cm}^2$ are related to their different non-thermal motion thresholds^15^. Between the thresholds for the formation of the particles and their motion, only those adatom and vacancy clusters are visible by STM that are formed by chance so close to the surface that their excess energy is sufficient for a transport to it.

### Formation of vacancy-interstitial pairs

These processes happen despite the fact that our fluence regime is still an order of magnitude below ablation thresholds^13^ even for an infinite number of pulses. To understand their atomistic origin, we now turn to the unexpected formation of the vacancy-interstitial pairs in the bulk. The void formation discussed in the introduction above the ablation threshold was attributed to the relaxation of the laser-induced pressure caused by the temperature rise. This cannot be the origin of the surface restructuring at the moderate increase in temperature as calculated. Laser-induced melting and ablation are, for Cu, also discussed in terms of bond softening, where the removal of electrons from bonding states allows single atoms to leave the crystal^38^.

**Figure 9:**
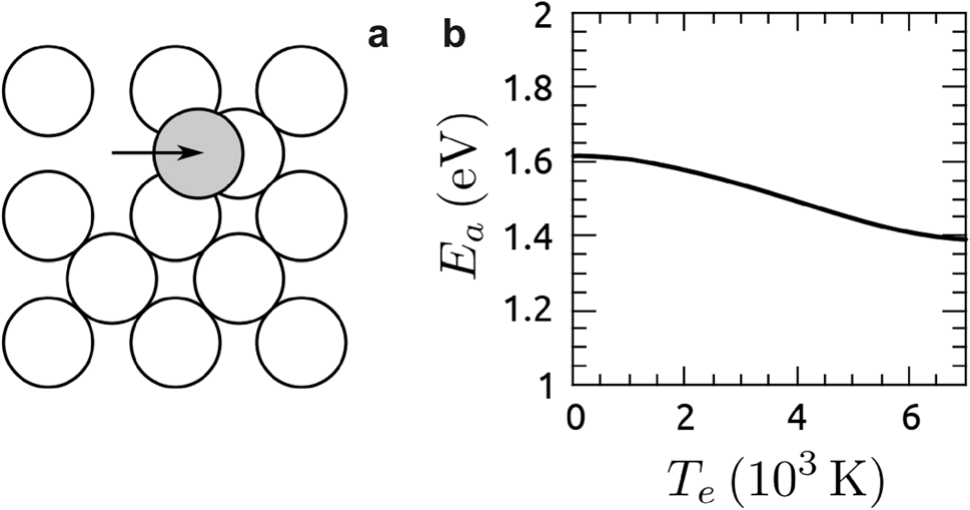
Bond softening: (**a**) scheme for modeled process (**b**) activation energy for process shown in panel (**a**) in dependence of electron temperature $T_e$.

Though for Ag a bond hardening is predicted^39^, we evaluate whether non-thermal bond softening plays any role in the process of surface roughening by computing the activation energy for a silver atom to hop from a surface site onto the terrace at different electronic temperatures (Fig. 9a). Using finite-temperature DFT and the nudged elastic band method^40^ to converge the minimum energy pathway, the activation energy for this process is around 1.6 eV at $T_e = 0~\hbox {K}$ (Fig. 9b). Increasing the electronic temperature to $T_e = 2000~\hbox {K}$ ($F = 1~\hbox {mJ/cm}^2$) reduces the barrier by only 0.05 eV. Even at 7000 K, the barrier is still around 1.4 eV. As this subtle bond softening lasts only for less than a picosecond, it seems unlikely that bond softening is responsible for the formation of surface vacancies. A similar conclusion is expected for the formation of vacancy-interstitial pairs in the bulk.

As the average energy is not sufficient to explain the observed processes, there must be some fluctuation, where more than the average energy is localized at one atom to form an interstitial-vacancy pair. This is possible in two scenarios. Either interstitial formation takes place before the laser excitation is converted into delocalized electron Bloch waves or these Bloch waves get localized. For the first scenario, short-lived excitons in *d*-band metals^41^ offer a possible pathway. Such transient exciton-like states are induced by ultra-short laser illumination of metal surfaces supporting surface and image potential bands^42^. They result from photo-excitation of 3*d* electrons to an empty *s* band of an atom (cf.^43^) and trapping of the excited electrons by the 3*d* holes^41,44^. If the lifetime of the transient exciton was long enough to couple to nuclear degrees of freedom of an individual atom in the bulk, the formation of vacancy-interstitial pairs would be possible. This scenario would be similar to the DI(M)ET (dynamics induced by (multiple) electron transitions) employed to explain femtochemistry. The energy of such an excited atom would be sufficient for bond cleavage to form an interstitial. For the second scenario, we propose that initial interstitials are formed via an electron or hole localizing at preexisting optically active defects, intrinsic to each crystal, in particular to soft metals as Ag. In subsequent laser pulses, those interstitials and vacancies that are optically active act as additional defects for localization. At present, our experimental results are awaiting a clear explanation. Future developments in theoretical methodology might confirm one of these scenarios to inspire further discussions about these novel phenomena.

### Generalization of the results

**Figure 10:**
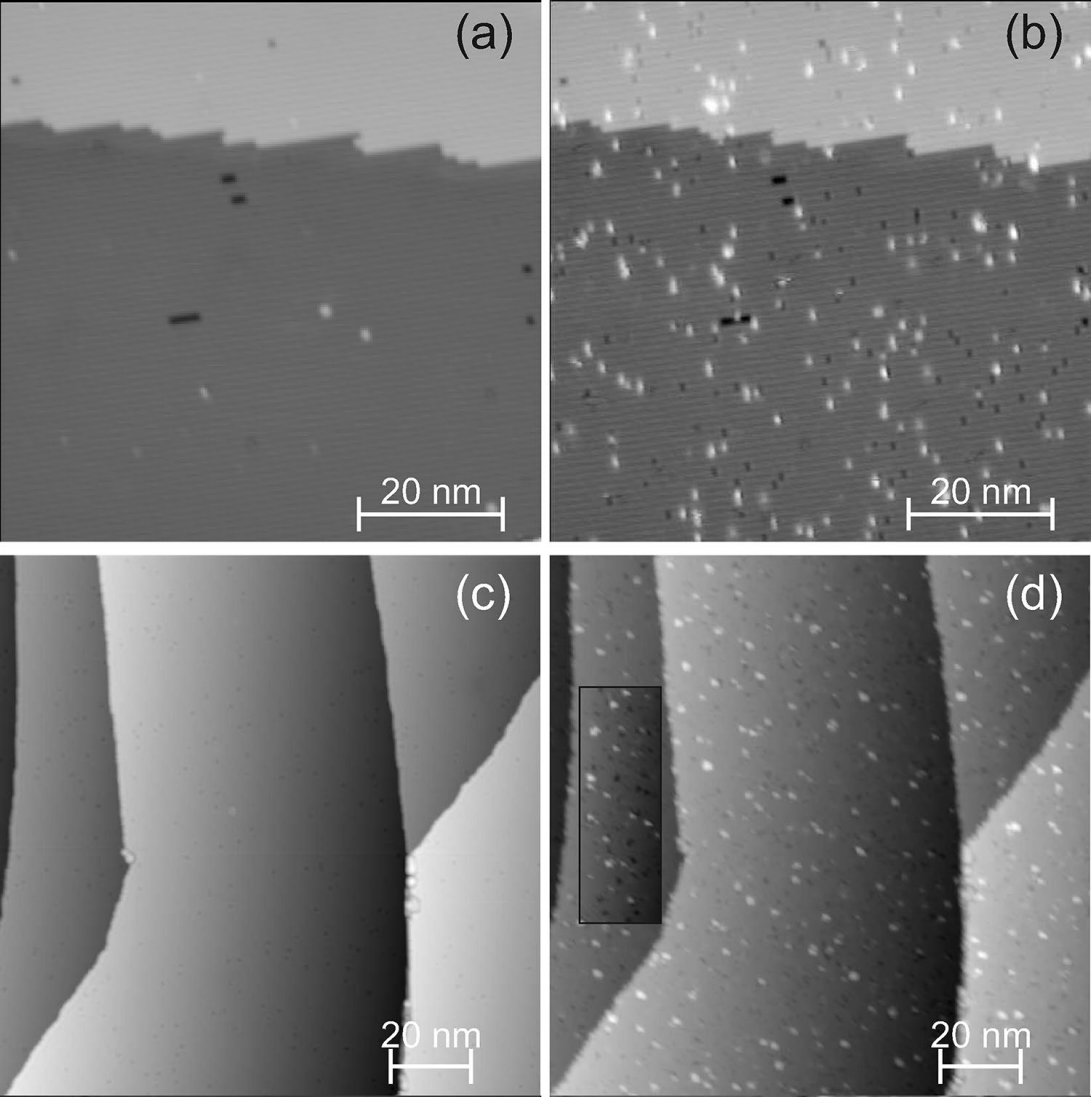
Effect of low-intensity laser illumination on Cu surfaces: (**a**,**b**) STM images of Cu(511) before (**a**) and after (**b**) illumination at an absorbed SPP fluence of $0.2~\hbox {mJ/cm}^2$, $10^6$ laser pulses, − 200 mV (**a**) 500 pA, and (**b**) 10 pA (**c**,**d**) STM images of Cu(111) before (**c**) and after (**d**) illumination at an absorbed SPP fluence below $5~\hbox {mJ/cm}^2$, $10^7$ laser pulses, 228 mV, 100 pA; contrast is increased in rectangle.

Silver was chosen for this in-depth analysis because of its weak electron–phonon coupling and strong electron thermal conductivity, which will show more evident traces of structural changes in comparison to transition metals because of the long lifetime of the excitons due to slow screening. These properties also lead to a lower maximum phonon temperature, which makes thermal diffusion effects more unlikely. However, the reasoning above suggests that effects are expected at other metals, only the quantitative details of fluence dependencies and thresholds are expected to shift. For confirmation, we repeated the experiment on two Cu surfaces, Cu(511) and Cu(111). Cu(511) is a surface that is vicinal to Cu(100), exhibiting thus the same surface geometry. The 0.63 nm wide terraces on Cu(511) show up in STM images as stripes (Fig. 10a). Also in this case, holes and protrusions of different sizes cover the surface upon laser illumination (Fig. 10b), validating that the creation of adatom and vacancy clusters is not specific to one material. Moreover, the secondary step edge at the top of the image in Fig. 10a,b remains unaltered, corroborating that the additional material does not originate from step edges.

Also on the Cu(111) surface holes and adatom clusters are induced by laser illumination (Fig. 10c,d). Their creation confirms that the observed phenomenon is not restricted to (100) facets. As compared to the tiny clusters on Ag(100) or Cu(511), the clusters are more extended on average. We trace this difference to a smaller laser-induced surface diffusion barrier, consistent with the lower thermal diffusion barriers on (111) as opposed to (100) faces of fcc crystals. Thus, different types of clusters could be induced in dependence on the facet choice. Moreover, clusters of this non-equilibrium shape would not develop in homo-epitaxial growth, even at low temperature^45^.

## Conclusion

We conclude that ultra-short laser pulses significantly alter the surface texture by forming adatom and vacancy clusters far below the laser melting thresholds. We trace these changes to vacancy-interstitial formation and motion of these bulk particles to the surface, both in an electron-induced process. Different thresholds for their formation provide the unique opportunity to form surfaces of desired texture. The surface modification at sub-melting fluences might reduce the energy density necessary for laser-induced melting and thus enlarges our fundamental understanding of non-equilibrium light-matter interactions.

Our study opens up further interesting perspectives. It is well established that turnover rates of surface reactions change dramatically with slight changes to the surface structure^46,47^. The increase in turnover rates due to specifically formed particles has been demonstrated recently^48^. Thus, any femtochemical reaction that operates at comparable fluence will profit from the roughening of the surface. Indeed, our results suggest that the surface can hardly be assumed static in such reaction experiments.

This surprising result will influence the understanding of all processes induced by ultra-short laser illumination, including, but not limited to, laser melting, ablation, and femtochemistry. In future, surface design will profit from the understanding of the microscopic mechanisms occurring under the highly non-equilibrium conditions formed in the near-surface region of a metal by ultra-short laser illumination.

## Supplementary information

Supplementary Information.
