# Supplementary material for: Real-space observation of surface structuring induced by ultra-fast-laser illumination far below the melting threshold
Source: Sci Rep. 2021 Jun 24;11:13269. doi: 10.1038/s41598-021-91894-w (PMC8225848; doi:10.1038/s41598-021-91894-w)
Supplement: Supplementary file 1 — Supplementary Information. [file 41598_2021_91894_MOESM1_ESM.pdf]

Supporting online material: Surface structuring induced by ultra-fast-laser illumination far below the melting threshold

Ch. Zaum[1], N. Osterloh[2], R. Darkins[3], D.M. Duffy[3], K. Morgenstern[2]

[1]*Leibniz Universität Hannover, Institut für Festkörperphysik,*
Abteilung für atomare und molekulare Strukturen (ATMOS),
Appelstr. 2, D-30167 Hannover, Germany

[2]*Ruhr-Universität Bochum, Lehrstuhl für*
physikalische Chemie I, D-44780 Bochum, Germany

[3]*Department of Physics and Astronomy and London Centre for Nanotechnology,*
University College London, Gower Street,
London WC1E6BT, United Kingdom.

(Dated: April 26, 2021)

Abstract

This supporting material gives some literature overview of the exponents in the power dependence of the yield of femtochemical reactions on fluence. Step edges are compared before and after laser illumination. Finally, the sizes of the created adatom clusters are analyzed for different fluences.

TABLE I: Fluence dependence in fs-laser induced experiments

process	system	photon energy	pulse length	absorbed single-pulse peak fluence	exponent c	reference
desorption	CO/Ru(0001)	800 nm/1.5 eV	110 fs	$\approx 4 - 140$ mJ/cm^2	2	[2]
oxidation	CO/Ru(0001)	800 nm/1.5 eV	110 fs	$\approx 4 - 140$ mJ/cm^2	2	[2]
desorption	NO/Pd(111)	n/a	200 fs	modeling	7.2	[3]
dephasing of						
stretching vibration	Cs/Pt(111)	800 nm/1.5 eV	150 fs	$10 - 13$ mJ/cm^2	2.2	[4]
desorption	15 BL H_2O/Pt(111)	800 nm/1.5 eV	130 fs	$14 - 20$ mJ/cm^2	6	[5]
diffusion	O/vicinal Pt(111)	800 nm/1.5 eV	55 fs	$6 - 14$ mJ/cm^2	15	[6]
desorption	NO/Pd(111)	620 nm/2 eV	200 fs	$2.4 - 6.4$ mJ/cm^2	3.3	[7]
desorption	O_2/Pt(111)	800 nm/1.5 eV	80 fs	$6 - 36$ mJ/cm^2	6.5 ± 0.4	[8]
and reaction	CO_2/Pt(111)	800 nm/1.5 eV	80 fs	$6 - 36$ mJ/cm^2	6.5 ± 0.4	[8]
desorption	O_2/Pt(111)	400 nm/3.1 eV	80 fs	$6 - 36$ mJ/cm^2	3.8 ± 0.5	[8]
and reaction	CO_2/Pt(111)	400 nm/3.1 eV	80 fs	$6 - 36$ mJ/cm^2	3.8 ± 0.5	[8]
desorption	O_2/Pt(111)	310 nm/4 eV	110 fs	$01 - 4$ mJ/cm^2	6.3 ± 0.5	[9]
oxidation	CO/Pt(111)	620 nm/2 eV	90 fs	$1 - 4$ mJ/cm^2	6.3 ± 0.5	[9]
associative						
desorption	H_2/Ru(0001)	n/a	n/a	$16 - 22$ mJ/cm^2	2.8	[10]
associative						
desorption	D_2/Ru(0001)	n/a	n/a	$16 - 22$ mJ/cm^2	3.2	[10]

FLUENCE DEPENDENCE DURING FEMTOCHEMISTRY

Reactions induced by ultra-short pulse laser illumination are called femtochemistry [1]. The experiments are used to resolve molecule dynamics at surfaces on the sub-picosecond time scale. Above a threshold of a few mJ/cm^2, the non-adiabicity of a reaction is deduced from a power law fluence dependence of the reaction yield, $Y \propto F^c$. The experiments show surprisingly diverse exponents ranging from 2 to 15 (Tab. I).

Step edges during laser illumination

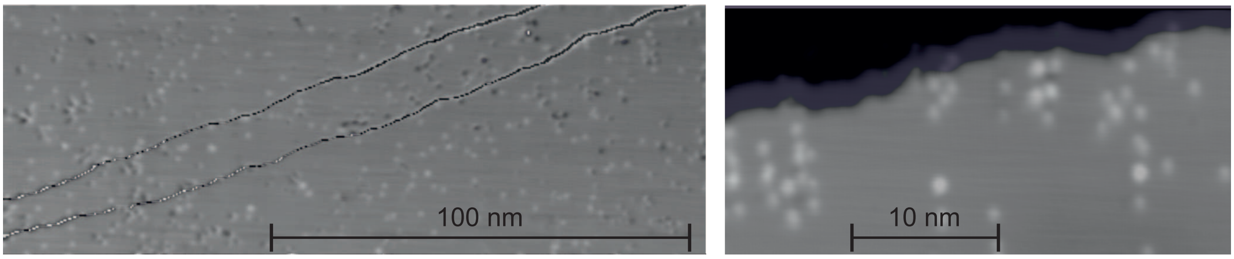

FIG. 1: Step edges on Ag(100) during laser illumination at a SPP fluence of 85 μJ/cm^2, 10^7 pulses, 113 mV, 81 pA: (a) Images with cyclic contrast are superimposed semi-transparently with inverted contrast to the image before laser illumination (b) Image with normal contrast are superimposed semi-transparently with the image after laser illumination vertically displaced downwards.

The step edges are not at the origin of the additional material. This statement is based on a detailed analysis of several ten step edges at different SPP fluences. Unfortunately, it is intrinsically difficult to visualize changes to STM images at step edges in static images. We thus show two different approaches in Fig. 1. In both cases, the step edges before and after illumination deviate slightly in the imaged orientation due to the unavoidable piezo creep, but otherwise they follow exactly the same trace. Occasionally an additional cluster appears close to the step edge, but we never observe a retracted step edge. In view of the amount of additional material that covers the surface, it can thus not originate from the step edges. This direct comparison is made possible by our ability to re-image the same spot of the surface before and after illumination.

Analysis of adatom cluster sizes

To quantify the non-monotonic fluence dependencies of the created adatom clusters, we analyze their size at different SPP fluences, between the thresholds for the vacancy-interstitial formation and vacancy motion (Fig. 2a), between the thresholds for the vacancy and adatom motion (Fig. 2b), and above the threshold for adatom motion (Fig. 2c). Mainly small adatom clusters with a perimeter length of up to 0.5 nm and a few in the range of 2 nm are produced at a low SPP fluence of $\sim$ 63 μJ/cm^2. At the same fluence, larger adatom clusters disappear (Fig. 2a). This is direct proof that bonds can be broken despite the very

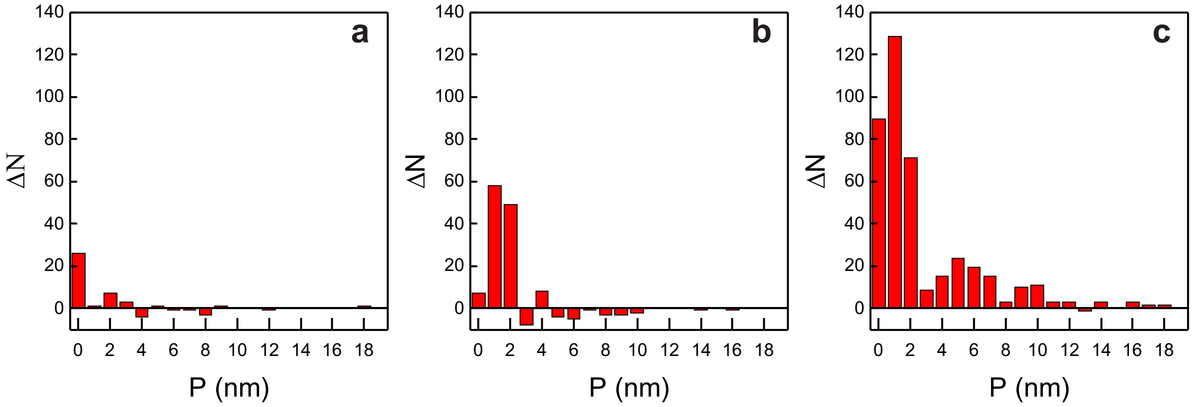

FIG. 2: Number of additional clusters ΔN of a size with perimeter P on an area of 4500 nm^2 on Ag(100), 10^7 pulses at a SPP fluence of (a) 66 μJ/cm^2 (b) 130 μJ/cm^2 (c) 185 μJ/cm^2.

moderate transient thermal heating by the laser. More adatoms clusters with perimeters up to 2.5 nm are created at an intermediate SPP fluence of $\sim$ 130 μJ/cm^2 (Fig. 2b). Also at this SPP fluence, the dissociation of a few larger adatom clusters leads to smaller adatom clusters. Only at a SPP fluence above 180 μJ/cm^2, the number of appearing adatom clusters outnumbers the disappearing ones at almost all perimeters. At this fluence, additional adatom clusters must result from the newly created interstitials in the bulk emerging to the surface.

Note that the size distribution changes non-monotonously with perimeter size (Fig. 2c). Also at the highest fluence, clusters in the range up to 2.5 nm are preferred. Other preferred sizes have perimeters peaking around 5 nm and 9 to 10 nm. This observation suggests that the larger clusters agglomerate from smaller clusters indicating their laser-induced mobility on the surface.

---

[1] C. Frischkorn, M. Wolf, Femtochemistry at metal Surfaces: nonadiabatic reaction dynamics. Chem. Rev. 106, 4207-4233 (2006).

[2] M. Bonn, S. Funk, Ch. Hen, D.N. Denzler, C. Stampfl, M. Scheffler, M. Wolf, G. Ertl, Phonon-versus electron-mediated desorption and oxidation of CO on Ru(0001). Science 285, 1042-1045 (1999).

[3] J.A. Misewich, T.F. Heinz, D.M. Newns, Desorption induced by multiple electronic transitions.

Phys. Rev. Lett. 68, 3737-3740 (1992).

[4] K. Watanabe, N. Takagi, Y. Matsumoto, Direct time-domain observation of ultrafast dephasing in adsorbate-substrate vibration under the influence of a hot electron bath: Cs adatoms on Pt(111). Phys. Rev. Lett. 92, 057401 – 1-4 (2004).

[5] E.H.G. Backus, M.L. Grecea, A.W. Kleyn, M. Bonn, Ultrafast electron-induced desorption of water from nanometer amorphous solid water films. J. Phys. Chem. B 111, 6141-6145 (2007).

[6] H. Ueba, M. Hayashi, M. Paulsson, B.N.J. Persson, Adsorbate hopping via vibrational-mode coupling induced by femtosecond laser pulses. Phys. Rev. B 78, 113408 – 1-4 (2008).

[7] J.A. Prybala, T.F. Heinz, J.A. Misewich, M.M.T. Loy, J.H. Glownia, Desorption induced by femtosecond laser pulses. Phys. Rev. Lett. 64, 1537-1540 (1990).

[8] S. Deliwala, R.J. Finlay, J.R. Goldman, T.H. Her, W.D. Mieher, E. Mazur, Surface femtochemistry of O_2 and CO on Pt(111). Chem. Phys. Lett. 242, 617-622 (1995).

[9] D.G. Busch, W. Ho, Direct Observation of the crossover from single to multiple excitations in femtosecond surface photochemistry. Phys. Rev. Lett. 77, 1338-1341 (1996).

[10] A.C. Luntz, M. Persson, S. Wagner, C. Frischkorn, M. Wolf, Femtosecond laser induced associative desorption of H_2 from Ru(0001): Comparison of "first principles" theory with experiment. J. Chem. Phys. 124, 244702 – 1-9 (2006).
